# Fit to fight – from military hygiene to wellbeing in the British Army

**DOI:** 10.1186/s40779-020-00248-6

**Published:** 2020-04-07

**Authors:** Martin C. M. Bricknell, Colonel David A. Ross

**Affiliations:** 1 Health and Military Medicine, Conflict and Health Research Group, School of Security Studies, King’s College London, Strand Campus, London, WC2R 2LS UK; 2 Parkes Professor of Preventive Medicine, Robertson House, Camberley, GU15 4NA UK

**Keywords:** Public health, Hygiene, Occupational medicine

## Abstract

This paper reviews the historical evolution of the language and organization surrounding the health of personnel in the British Army from ‘hygiene’ through  to ‘wellbeing’. It starts by considering the health of the army in the mid-nineteenth century and the emergence of military hygiene as a professional subject. It continues by looking at advances in military hygiene in the two world wars. Hygiene was replaced by the term ‘health’ in the 1950s as the collective noun used by professionals working in this field. This unity split when the professions of occupational medicine and public health established separate faculties and training pathways. However, the health issues for the armed forces remain fundamentally unchanged. Going forward, the term ‘wellbeing’ is helping to refresh the close relationships between executives, their medical advisers and those within the population of health professions charged with keeping the British Army healthy. The core theme is the collaborations between civil society, executive leadership and medical services in maximizing the health of the military population from recruitment through to life as a veteran.

## Background

This paper reviews the evolution of the conceptual framework for sustaining the health of the British Army as an occupational group from the introduction of military ‘hygiene’ in the middle of the nineteenth century to that of ‘wellbeing’ in the early twenty-first century. This evolution is based on the story of the British Army. However, this does not mean that a similar story is less important in either the Royal Navy or the Royal Air Force. Indeed, since the establishment of a tri-service Surgeon General in 1990, this narrative has become framed around the health of armed forces personnel. This description of the evolution of thinking about the health of a defined population mirrors the evolution of the thinking and language used for global health, especially from a ‘hygienic’ approach to protecting the health of a population, through to  the improvement of health by social and political interventions alongside the improvement of health services [1].

The historical journey starts with the emergence of the speciality of military hygiene and its influence on the public health movement in the United Kingdom. Next, the current study will consider the public health innovations that arose from strategic concern over the quality of the nations’ manpower for the army prior to World War I. Experiences in World War I and World War II led to the emergence of ‘health’ as an attribute for military capability based on a holistic perspective of the performance of soldiers within a physical and social environment. This holistic view of ‘army health’ lasted until the early 1970s, when civilian industrial medicine and social medicine became separate medical specialities of occupational medicine and community medicine (and later of public health). This separation split the professional training of specialist army health doctors into either occupational medicine or public health. Since the 1990s, medical services have become more Joint and Defence in their orientation. The health of the armed forces continues to be of strategic importance, and the current paper will close by discussing the emergence of thinking about ‘health and wellbeing’ for armed forces personnel and veterans through collaborations across the government, between the armed services and within healthcare professions. The core theme of the current study is the collaboration between civil society, executive leadership and medical services in maximizing the health of the military population from recruitment through to life as a veteran.

## Military health and hygiene in the nineteenth century

The story of the ravages of disease experienced by the British Army during the Crimean War (1853–1856) and the role of Florence Nightingale to bring this experience to the attention of the British government and the public is widely recognized. Less well known is the impact of disease on the army in garrisons both abroad and in England. In 1858, a Royal Commission led by Sidney Herbert (Secretary of State for War and a close friend of Florence Nightingale) published a report on the health of the army. This report showed that the mortality of soldiers stationed in England was 17.5 / 1000 people-year, which was substantially greater than the mortality rate of the general adult male population at 9.2 / 1000 people-year [2]. Even more surprising, the mortality of the army when garrisoned in England was nearly one-third greater than that of the army when it was stationed at Sevastopol, Crimea, in 1856 (at 12.5 / 1000 people-year). It was demonstrated through statistical analysis that substantial savings in army manpower could be attained by improving the health of the army through better hygiene, better army hospitals and better-trained army doctors. The report made wide-ranging recommendations for the improvement in the organization and management of the Army Medical Services, army hospitals, and the education of army medical officers in military medicine and hygiene. This report made the health of soldiers into a political and executive issue rather than solely a medical issue.

## Edmund Parkes - the first professor of military hygiene

There was a substantial reform of the Army Medical Services over the course of the second half of the nineteenth century. This reform included the creation of the Army Medical School in 1860 at Fort Pitt in Chatham (which became the Royal Army Medical College, Millbank, in 1907). The school was endowed with three professors: one of clinical and military medicine, one of clinical and military surgery, and one of sanitary science and military hygiene. The appointment of Edmund Parkes as the first Professor of Military Hygiene became one of the most important innovations in the education of military medical personnel. Parkes joined the army at the age of 22 and served for 3 years before establishing himself in private practice in London and University College. In 1855, he was sent to Turkey and established a military hospital at Renkoi based on a prefabricated structure that was designed and manufactured by Isambard Kingdom Brunel [3].

After his appointment, Parkes wrote the Manual of Practical Hygiene that had influence across the public health movement in the UK and overseas [4]. The manual’s preface highlighted the altered position of the army medical officer because of changes to the Queen’s Hospital regulations in 1859 [5]. *‘Previously, the Army Surgeon had been entrusted officially merely with the care of the sick…..(now) he is ordered to advise commanding officers in all matters affecting the health of troops, whether as regards garrisons, stations, camps and barracks, or diet, clothing, drill, duties or exercises’*. This is the epitome of occupational medicine. In the introduction, Parkes defines hygiene as the *‘..art of preserving health; that is, of obtaining the most perfect action of body and mind during as long a period as is consistent with the laws of life. In other words, it aims at rendering growth more perfect, decay less rapid, life more vigorous, death more remote’*. This is the epitome of public health.

The introduction continues by stating that *‘..in many cases, the employer of labour finds that, by proper sanitary care of his men, he reaps at once an advantage in better and more zealous work, in fewer interruptions from ill-health so that his apparent outlay is more than compensated. This is shown in the strongest light by the Army. The State employs a large number of men, whom it places under its own social and sanitary conditions. It removes from them much of the self-control with regard to hygienic rules which other men possess and is therefore bound by every principle of honest and fair contract to see that these men are in no way injured by its system. But more than this: it is as much bound by its self-interest. It has been proved over and over again that nothing is so costly in all ways as disease and nothing is so remunerative as is the outlay which augments health, and in doing so, augments the amount and value of the work done’*. These three quotes provide enduring social and economic arguments for protecting the health of soldiers in the army and show the interrelationship between the modern clinical specialities of occupational medicine and public health in advising on the health of army personnel.

This focus on military hygiene reduced all-cause admissions to hospitals per thousand strength from 1060 in the 1870s to 1020 in the 1880s, 850 in the 1890s, and 500 in the first decade of the twentieth century [6]. By the time of his death in 1876, Parkes had established the importance of hygiene within the military and of the contribution of military hygiene experts within the wider public sector alongside the civilian Medical Officers of Health in towns and cities. Teaching military hygiene now had equal status to military medicine and surgery.

## The health of the public and its impact on the health of the Army

The army faced many challenges during the Boer War in South Africa (1899–1902) both in trauma care and preventive medicine. However, the requirement to mobilize the manpower of Great Britain for the war also exposed the very poor standards of health amongst the civilian male population. This resulted in very high rates of rejection for military service. Such was the concern that Parliament set up a Committee on Physical Deterioration *‘to determine, with the aid of such counsel as the medical profession are able to give; the steps that should be taken to furnish the Government and the Nation at large with periodical data for an accurate comparative estimate of the health and physique of the people; to determine generally the causes of such physical deterioration as does exist in certain classes; and to point out the means by which it can be most effectually diminished’* [7]. This report made 53 recommendations, many of which are recognizable as public health and industrial health improvements that persist today. These recommendations included a national anthropometric survey of the population; the medical examination of school children, factory workers and coal miners; the training of mothers in the domestic economy (which became Health Visiting); and health education and sport as part of the school curriculum. The army continued with the progress of sanitary reform after the Boer War. In 1904, training in basic hygiene was added to the curriculum for officer training, and the Army School of Sanitation was established in 1906. There was considerable technical progress in subjects such as water purification, the ‘hygiene of the march’ and immunization against infectious disease [8]. Institutional knowledge was codified as the Manual of Elementary Military Hygiene, published by the War Office in 1912 [9]. Thus, by the beginning of World War I, the public health movement had unified the importance of both the health of the population to be recruited into the army and the maintenance of the health of soldiers once in the army.

## World War I – hygiene at scale

The substantial role played by military hygiene in keeping the mobilized armed forces fit for military operations during World War I was summarized in 1924 by Colonel Anderson, who was a professor of hygiene at the Royal Army Medical College [10]. This experience led to a greater understanding of infectious diseases such as typhus, relapsing fever, cerebrospinal fever, and influenza and the prevention of these diseases by setting standards for accommodation, sanitation and cleanliness. These standards also included the education and training of individual soldiers in ‘sanitary habits’ [11]. Many aspects of military hygiene were transferred to civil society. The work to define the food and energy requirements for soldiers on the march was used by the Food (War) Committee of the Royal Society to determine the rationing requirements for the civil population. The requirement for preserved military rations led to the development of pasteurization and canned food. The subject of medical topography, which we would now call travel health, emerged from the requirement to analyse the potential impact of endemic medical disease on military forces. Finally, the Chemical Warfare Medical Committee, under the chairmanship of the first Director of Military Hygiene, Colonel Sir William Horrocks, supervised the development of protective equipment and medical treatments for gas warfare, which could be considered a very specific branch of industrial hygiene. This momentum was maintained during the inter-war years through the creation of an Army Hygiene Directorate in 1919 and an Army Hygiene Advisory Committee comprised of military and civilian experts. In 1922, a hygiene specialist was appointed to the staff of the Army School of Physical Training to carry out research and advise on the physiology of exercise [12]. This knowledge was consolidated through the publication of the Manual of Army Hygiene and Sanitation in 1934 [13].

In his presidential address of 1922 to the Navy, Army and Air Force groups of the civilian Society of Medical Officers of Health, Major General Sir William Macpherson [14] summarized military hygiene as *‘the maintenance of physical fitness, physical training, the hygiene ‘of the march’, the relationship between food and energy and camp sanitation*’. He also highlighted other subjects in military hygiene that have equal importance in civilian life, such as the prevention of epidemics and the preservation of health in communities living in close contact with one another. Major-General Beveridge [15], in his presidential address to the same body a year later, compared military hygiene and public health as follows: *‘Public health, as applied to the civil community, is concerned with the individual during the whole period of life, but in the fighting services it is chiefly concerned, for all practical purposes with selected personnel during a certain period of life’*. This close relationship between the practice of hygiene in the army and civilian practice by members of the Society of Medical Officers of Health continued through the twentieth century as the professional body evolved into the Faculty of Community Medicine in 1973.

## World War II – hygiene to health

In World War II, military hygiene continued to be critically important in ensuring the health of the army both in the UK and in operations across the world, notably extending from physical health to mental and social health. There were advances in the application of science to hygiene, for example, the introduction of chemoprophylaxis against malaria, the use of dichloro-diphenyl-trichloroethane (DDT) as an insecticide, and the use of penicillin in the control of sexually transmitted disease [16]. Lessons from the contribution of hygiene to winning the campaign in the Middle East were incorporated into the preparation of the 21st Army Group to fight in Northern Europe. Field Marshall Montgomery is quoted as saying that *‘the men of 21 Army Group were fully immunised and fully trained; their morale was at its highest; they were well clothed and well fed; they were fighting in a climate to which the average British soldier is well accustomed; hygiene, both personal and unit, was exceptionally good; welfare services were well organised. The exhilarating effect of success also played a role in reducing rates of sickness’* [17].

The opening section of the chapter on the Army Medical Services in the volume of Principal Medical Lessons of the Second World War describes how the concept of the nature of health was enlarged to encompass much more than just the physical functioning of the single individual. ‘*It came to be recognised that disharmony between the individual and the conditions and circumstances that obtained within the community was the cause of much ill-health and so the search for causation became extended from the physical to the social environment of individuals and groups’* [18]. This social perspective on health was also strongly championed by General Sir Ronald Forbes Adam, the Adjutant-General (head of personnel for the British Army), alongside medical services. He directed the development of aptitude selection based on psychological tests and physical tests and introduced the concept of the demobilized soldier as a returning citizen member of the nation. The experience of preventive medicine in World War II also re-emphasized the responsibilities of commanders to ensure that the recommendations of their medical advisers were implemented alongside the developments in hygiene measures by the Army Medical Services [19].

## Army Health – 1950s - organization

In addition to the widening of the outlook in the attainment of physical and mental health in the British Army, it was also felt that the term ‘hygiene’ had become restricted to the field of sanitation rather than the earlier broad ‘health’ perspective. Therefore, in 1950, the Directorate of Army Hygiene became the Directorate of Army Health [20] within the Army Medical Directorate of the War Office. The Army Health Advisory Committee (comprising leading civilian authorities on public health, malariology, physiology, nutrition, and statistics) continued to provide external advice. The directorate was responsible for leading the Army Health Organisation [21]. Senior staff officers were graded as specialists in Army Health with civilian public health qualifications. The majority also attained a Diploma in Tropical Medicine and Hygiene that was jointly taught by the Royal Army Medical College and the London School of Hygiene and Tropical Medicine. Postgraduate education for medical officers in Army Health was delivered through the Army Health Department of the Royal Army Medical College, and non-professional training was provided by the Army School of Health to members of the Royal Army Medical Corps (RAMC), non-RAMC officers and other ranks. King’s Regulations and Army Council Instructions continued to place the primary responsibility for the health of the army upon the chain of command. Senior leaders of medical services framed this as the welding together of the traditional art of man-management and the modern scientific methods of disease control into the so-called ‘health discipline’ [22].

Organizing health education was one of the primary tasks of specialists in Army Health. To aid them in this task, new films dealing with personal and communal hygiene were released in 1950, and Mobile Health Training Teams were set up. A great deal of thought was given to the various educational techniques involved. A new pamphlet was produced to help the individual soldier, Your Health and You [23]. The lessons of World War II were incorporated into the Handbook of Army Health [24] for non-specialists and into the Manual of Army Health for specialists [25]. The importance and interdisciplinary nature of Army Health continued to be championed during the 1960s. In an article in the *Journal of the Royal Army Medical Corps*, Colonel Lewis (Professor of Army Health) stated that *‘to select military personnel in accordance with high physical and mental standards, to subject them to long and expensive training, and then to dissipate a large portion of this human treasure in non-productive man-hours wasted through preventable ill-health is uneconomical, to say the least; and, when manpower resources are limited the issue is nationally vital (…) the successful practice of Army Health calls for team-work in which everyone in the Army, irrespective of regiment, corps, trade; grade, mode of employment and rank has a part to play’* [26]. These observations echo the social and economic arguments about military hygiene made by Parkes almost a century earlier.

## Army Health separation into Public Health and Occupational Medicine in the 1970s

This integrated Army Health Organisation continued until the late 1970s, when postgraduate professional training for medical officers in the RAMC was formally structured into general practice, hospital specialities and a new grouping, namely, Army Community and Occupational Medicine (ACOM). Doctors in this third stream were required to qualify for Membership of the Faculty of Community Medicine or Occupational Medicine [27]. ACOM changed to Army Public Health and Occupational Medicine (APHOM) when the Faculty of Community Medicine was renamed the Faculty of Public Health in 1989 [28]. Slowly, the professional training routes for Army Health doctors split, despite the considerable overlap of their roles as preventive medicine or health specialists [29]. Consultants in each speciality were quite public in their views of the differences in their professional knowledge [30]. The postgraduate medical training route for both specialities followed the discrete civilian faculty models with a blend of military and civilian experience [31]. This led to the demise of the dual-speciality education course at the Royal Army Medical College and the competition to label the previous health posts as either public health or occupational medicine [32]. An Army Health research capability was created by the formation of the Army Personnel Research Establishment in 1965 [33]. This developed into the Army Occupational Health Research Unit in the 1980s that was manned by occupational physicians. It was disestablished when QuinetiQ was formed from the Defence Evaluation and Research Agency in 2001 with its military manpower being incorporated into the Army Health Unit in the Army Medical Directorate in the early 2000s.

Occupational medicine in the military has become a more clinical speciality, as army general practitioners and hospital specialists have become less closely associated with army personnel in their workplace [34]. This has also led to the creation of military occupational health (OH) nurses in a model that partially mirrors civilian practice. Public health has become a central staff function, with posts in the Joint Medical Group and the service commands [35]. This separation of medical specialties has also distanced the environmental health care from a ‘military health’ identity. Indeed, a review of occupational health in the armed forces published in 2009 did not mention the relationship between occupational health and the specialities of public health or environmental health [36]. Thus, the unifying identity of ‘Army Health’ became disaggregated during the 1980s into the early 2000s as the individual professions aligned to their separate identities in the civilian sector.

## Current issues in military health and wellbeing across the military life course

Many issues in military health remain the same as those of the nineteenth century, though the context has changed (and deaths in the barracks are very rare!). The 2017 Francois Report of a review of recruiting for the armed forces stated, *‘At present, over 90% of individuals who are failed when attempting to join the Armed Forces do so on medical grounds* [37]*’.* This has raised concerns about the physical and mental health of the recruitable population and the thresholds at which those with a pre-existing medical condition are excluded. At the other end of military service, the rate of medical discharge in the armed forces attracts attention because the two primary causes, musculoskeletal injury and mental ill health, are perceived to be preventable. Mental health in the armed forces and the veteran community has had particular political and media prominence, with the House of Commons Defence Committee conducting an inquiry into mental health in the armed forces and veterans from 2017 to 2019 [38]. Preventive medicine continues to be scrutinized, with legal cases pending on the anti-malarial Mefloquine, Q fever, non-freezing cold injury and noise-induced hearing loss.

The integration of professional knowledge from all health specialities to promote health and prevent disease for the military population has been re-emphasized over the last decade. The Defence Medical Services Top Structures review in 2008/2009 introduced a life-course approach to force health protection and the preparation of armed forces personnel for military operations. This starts at the stage of recruitment from the civil population through to understanding the long-term health effects of military service amongst Veterans and takes place on a ‘continuum of care’ [39]. The three services of the Royal Navy, the British Army and the Royal Air Force continue to promote wellbeing and healthy living through education during entry training and periodic mandatory additional training. Briefings on keeping healthy are included within pre-deployment training for military operations. There are active research programmes on physical fitness, diet, mental health and other dimensions of health in the military.

The military medical services remain empowered as advisers on health to the executives, with structural collaboration between the personnel and the medical function. In the evolution from ‘hygiene’ to ‘health’, the term ‘wellbeing’ has become preferable to that of ‘health’ to further emphasize the contribution of all other stakeholders in supporting armed forces personnel to maximize their physical, mental and social potential. The Defence Mental Health and Wellbeing Strategy, launched in 2017, described the role of the Defence Health and Wellbeing Board to bring coherence across Defence [40]. There are subordinate groups with the responsibility for lifestyles, injury prevention, preventive health and mental health. This shift is also occurring within civilian occupational medicine [41] and other military medical services [42]. In the army, the Director of Personnel for the Army is supported by a Senior Health Advisor (Army) as the most senior medical services officer. The Head of the Royal Navy Medical Service is the medical adviser to the Navy Board, and the Head of the Royal Air Force Medical Services is also the medical adviser to the Air Force Board [43]. Outside the Ministry of Defence, the National Health Service (NHS) Long-term Plan seeks to give people more control over their own health and to invest in more NHS prevention services [44]. The plan also includes a specific section on NHS services for armed forces personnel and veterans. The UK government has recently established an Office for Veterans Affairs to coordinate activities and existing funding to ensure that ex-service people get access to medical treatment, training and housing to meet their unique health needs [45].

## Conclusions

This paper has described the enduring importance of physical, mental and social health and wellbeing amongst army personnel as a key enabler of military capability. Although the language has changed (from ‘hygiene to ‘health’ and then to ‘wellbeing’), the core issues regarding the health of the British Army remain. The scourge of infectious disease in garrison in the 1800s has been addressed by improvements in housing, sanitation and wider public health measures such that mortality in the army population is no longer an issue. However, the quality of the health of the civilian population for entry into the army remains a concern, although the modern issue is obesity and poor physical conditioning rather than malnutrition. Non-medical, military leaders continue to emphasize the promotion and maximization of health for serving military personnel to support the personnel component of military capability. The current subject areas of lifestyles, injury prevention, preventive health and mental health are similar to those listed by Parkes in the late nineteenth century. The media and the wider public maintain an interest in how government services meet the health needs of the armed forces and military veterans. There has been a recent refreshing of the close relationship between military health and wider civilian public and occupational health. This paper has also demonstrated the requirement for a cadre of military health professionals with a combination of public health, occupational health and environmental health competencies who can provide technical advice that informs policies, procedures and practices in the promotion and protection of the health of the military population. This paper has focused on experiences in the UK, especially those of the British Army. It would be interesting to compare these results with other nations’ military medical experiences.

## Data Availability

Not applicable.

## Abbreviations

ACOM: Army Community and Occupational Medicine
APHOM: Army Public Health and Occupational Medicine
NHS: National Health Service
OH: Occupational health
RAMC: Royal Army Medical Corps
